# Ni_2_P/rGO/NF Nanosheets As a Bifunctional High-Performance Electrocatalyst for Water Splitting

**DOI:** 10.3390/ma13030744

**Published:** 2020-02-06

**Authors:** Jinyu Huang, Feifei Li, Baozhong Liu, Peng Zhang

**Affiliations:** 1 College of Chemistry and Chemical Engineering, Henan Polytechnic University, Jiaozuo 454000, China; mayfieldzzu@sina.com (J.H.); lifeifei@hpu.edu.cn (F.L.); bzliu@hpu.edu.cn (B.L.); 2 School of Electric and Information Egineer, Zhongyuan University of Technology, Zhengzhou 450007, China

**Keywords:** OER, HER, bifunctional, phosphate, graphene, Ni foam

## Abstract

The hydrogen generated via the water splitting method is restricted by the high level of theoretical potential exhibited by the anode. The work focuses on synthesizing a bifunctional catalyst with a high efficiency, that is, a nickel phosphide doped with the reduced graphene oxide nanosheets supported on the Ni foam (Ni_2_P/rGO/NF), via the hydrothermal approach together with the calcination approach specific to the hydrogen evolution reaction (HER) and the oxygen evolution reaction (OER). The Raman, X-Ray Diffraction (XRD), X-ray Photoelectron Spectroscopy (XPS), Transmission Electron Microscope (TEM), Scanning Electron Microscopy (SEM), High-Resolution Transmission Electron Microscopy (HRTEM), as well as elemental mapping, are adopted to study the composition and morphology possessed by Ni_2_P/rGO/NF. The electrochemical testing is performed by constructing a parallel two-electrode electrolyzer (Ni_2_P/rGO/NF||Ni_2_P/rGO/NF). Ni_2_P/rGO/NF||Ni_2_P/rGO/NF needs a voltage of only 1.676 V for driving 10 mA/cm^2^, which is extremely close to Pt/C/NF||IrO_2_/NF (1.502 V). It is possible to maintain the current density for no less than 30 hours. It can be demonstrated that Ni_2_P/rGO/NF||Ni_2_P/rGO/NF has commercial feasibility, relying on the strong activity and high stability.

## 1. Introduction

Hydrogen energy is an abundant and green energy with a high utilization rate and without secondary pollution, making it an ideal source of energy to replace fossil energy [1,2]. At present, electrochemical water decomposition is a key step in the production, storage and use of hydrogen, rechargeable metal air cells and fuel cells, which are widely regarded as a key step in efficient renewable energy [3,4,5,6]. At present, the most advanced catalysts for decomposing water include IrO_2_ for oxygen generation reaction (OER) together with Pt for the hydrogen evolution reaction (HER), with ~1.5 V reaching 10 mA/cm^2^ current for the entire water splitting [7,8,9]. Nevertheless, the application of these precious metals is restricted due to the high price and the scarcity.

Researchers have made lots of effort and have conducted studies in the effective OER and HER and the catalysts used contain a large amount of earth materials, like cobalt phosphate, transition metal dichalcogenides, perovskite oxides, transition metal oxides (TMOs), as well as nickel molybdenum alloy [10,11,12,13,14,15]. Even so, a huge difficulty in achieving a high performance of water splitting lies in applying the same catalyst as anode and cathode to synthesize the HER and OER catalysts in alkaline solutions [16,17,18]. The methods of straining, doping and other commonly used methods at present can lower the half reaction potential in an efficient manner [19,20]. In spite of this, these usually lead to a contradictory melting of two catalysts and as a result, the performance of the entire water splitting is weakened [21]. While according to the density functional theory calculation of nickel phosphide, the surface of Ni_2_P (001) at Ni and P sites is exposed and both the proton acceptor center and the hydride acceptor center exist to promote efficiency of hydrogen production by water splitting [22,23]. Therefore, nickel phosphide has a huge development potential.

Highly conductive materials such as metal, graphite, graphene, carbon black and carbon nanotube can be used for fabricating metal oxides with nanostructure for effectively collecting electron, thereby helping to enhance the electrical conductivity exhibited by electrons based on metal oxide [24,25,26]. Graphene is characterized by strong conductivity, electrochemical stability and flexibility, a high surface area as well as an outstanding mechanical performance and so forth, contributing to its wide application as a proper matrix for the development of metal oxides [27,28]. The graphene-based nanocomposites have the function of effectively using active metal oxides on the one hand and improving the mechanical strength as well as electrical conductivity exhibited by the resulting on the other hand.

**Figure 1:**
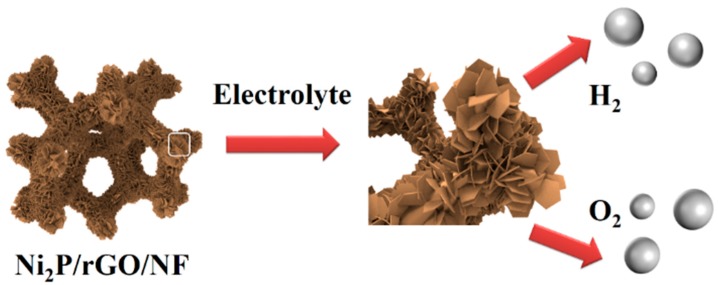
Map of overall water splitting in Ni_2_P/rGO/NF electrode.

In this work, we prepared the Ni_2_P doped with the reduced graphene oxide nanosheets array on the Ni foam (Ni_2_P/rGO/NF), firstly synthesizing the NiO doped with the reduced graphene oxide nanosheets array on the Ni foam (NiO/rGO/NF) by using the hydrothermal process, then synthesizing Ni_2_P/rGO/NF. Subsequently, a calcination approach was adopted after placing above prepared NiO/rGO/NF in the ceramic crucible, followed by the utilization of prepared catalysts for overall water splitting (Figure 1).

## 2. Experimental

### 2.1. Materials 

Graphene oxide (GO) sheets and nickel foam (NF) used in the study were obtained from the XFNANO Materials Tech Co., Ltd (Nanjing, China) and Shenzhen Green and Creative Environmental Science and Technology Co. Ltd. (Shenzhen, China), respectively. Purchased from Shanxi Kaida Chemical Co. Ltd (Shanxi, China), were 20 wt% Pt/C and 20 wt% IrO_2_. The Sinopharm Chemical Reagent Co. Ltd (Beijing, China) provided nickel nitrate hexahydrate (Ni(NO_3_)_2_·6H_2_O), hexamethylenetetramine, NaH_2_PO_2_, as well as Potassium hydroxide (KOH). All these chemicals had been used once they were received without any purification.

### 2.2. Ni_2_P/rGO/NF Synthesis

First, the NiO/rGO/NF was synthesized. Experimenters dissolved a certain amount of Ni(NO_3_)_2_·6H_2_O (5 mmol) and hexamethylenetetramine (5 mmol) in 30 mL ultra-pure water and constantly stirred the mixture, followed by the addition of 50 mg GO and 1 h of ultrasonication for the formation of a suspension. Subsequently, we transferred the resultant suspension together with pretreated Ni foam to a Teflonlined autoclave (50 mL) and heated it at 120 °C for 8 h. Second, the Ni_2_P/rGO/NF was synthesized. After the above prepared NiO/rGO/NF being placed in the ceramic crucible and the ceramic crucible with NaH_2_PO_2_ (1 mg) being placed on the upstream side of the furnace, 10 cm away from the NiO/rGO/NF received 2 h of calcination treatment at 300 °C (2 °C·min^−1^) under N_2_ flow.

### 2.3. Characterization and Electrochemical Measurements

The X-ray Diffraction (XRD) data were obtained from a RIGAKUD/MAX 2550 diffractometer (Rigaku Corporation, Tokyo, Japan) with a Cu Kα radiation (λ = 1.5418 Å). X-ray Photoelectron Spectroscopy (XPS) measurement was conducted on an ESCALABMK II XPS (VG Scientific, London, UK) taking Mg as the excitation source. Scanning Electron Microscopy (SEM) measurement was implemented on a XL30 ESEM FEG SEM (Carl Zeiss AG, Jena, Germany) under a 20 kV accelerating voltage. Transmission Electron Microscopy (TEM) measurement was conducted on a HITACHI H-8100 electron microscopy (Hitachi, Tokyo, Japan) under a 200 kV accelerating voltage. The electrochemical measurement was carried out on a CHI 660E electrochemical workstation relying on a standard three-electrode system. We used the above prepared electrode materials as working electrodes directly, taking the HgO/Hg (MOE) and the graphite rod as reference electrode and counter electrode, respectively. We converted all measured potentials to the reversible hydrogen electrode (RHE) following the Nernst equation, that is, E_RHE_ = E_Hg/HgO_ + 0.098 + 0.059 PH. The electrochemical test was implemented at 5 mV/s under the IR compensation. The electrochemical impedance spectroscopy (EIS) was implemented in the frequency (Hz) range of 1–1,000,000.

## 3. Results and Discussion

### 3.1. Physical Characterization

**Figure 2:**
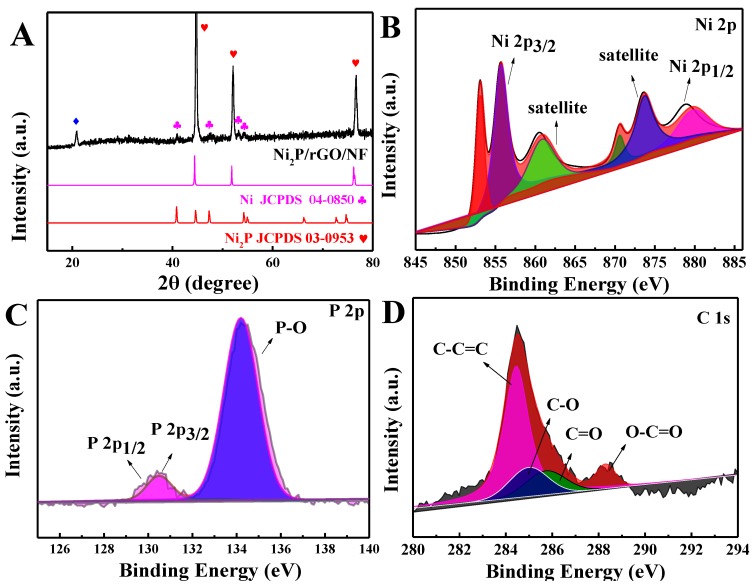
(**A**) X-ray Diffraction (XRD) of Ni_2_P/rGO/NF. X-ray Photoelectron Spectroscopy (XPS) of Ni 2p (**B**), P 2p 1s (**C**) and C 1s (**D**) for Ni_2_P/rGO/NF.

Figure 2A displays the XRD pattern about the Ni_2_P/rGO/NF. The diffraction peaks at 40.8°, 47.3°, 54.2°, 54.9° and 74.8°, corresponded to (111), (210), (300), (211) and (400) crystal planes of Ni_2_P (JCPDS No. 03-0953), respectively [29]. Besides, peaks of 44.4°, 51.7° and 76.5° well corresponded with the Ni (JCPDS No. 04-0850) [30]. However, the peak at 20.01° was caused by the reduced graphene oxide [31]. NiO/rGO/NF also well followed standard cards (Figure S1).

**Figure 3:**
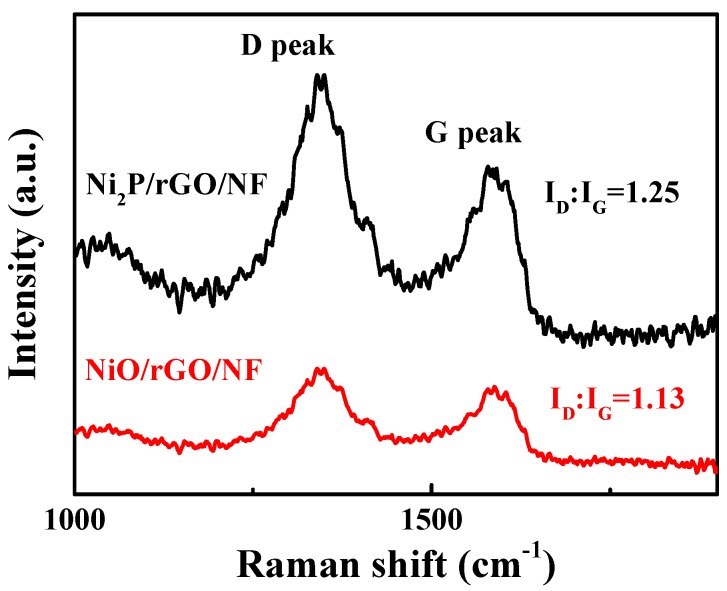
Raman of Ni_2_P/rGO/NF as well as NiO/rGO/NF.

The chemical composition as well as the valences were obtained via the XPS measurement. According to Figure 2B, peaks at the 853.4 eV and the 870.1 eV corresponded to Ni^2+^. Ni 2p_1/2_ at the 874.7 eV and the Ni 2p_3/2_ at the 857.7 eV may correspond to Ni^3+^ from surface oxide phase. Two satellite peaks at the 860.2 eV and the 879.1 eV stand for the oxidation state of the Ni^2+^ [32]. Based on Figure 2C, the P_3/2_ at 128.5 eV and P_1/2_ at 130.5 eV corresponded to P^2−^. The peak at 134.2 eV could belong to P-O from the surface oxide phase [29,33]. The C1s of Ni_2_P/rGO/NF (Figure 2D) have 3 components according with the C-C=C (284.3 eV), C-O (285.1 eV), C=O (285.6 eV), as well as O-C=O (287.9 eV). Raman spectra about the Ni_2_P/rGO/NF together with the NiO/rGO/NF composites are displayed in Figure 3. The peak at 1357 cm^−1^ accorded with D band and that at 1583 cm^−1^ accorded with G band. The G peak is related to the in-plane vibration of carbon atoms bonded to sp2, while the D peak is related to the electronic configuration of sp3 and vibration of carbon atoms and the I_D_:I_G_ intensity ratio is used to characterize the disordered degree of carbonaceous material [34]. Obviously, The I_D_:I_G_ values of Ni_2_P/rGO/NF and NiO/rGO/NF are 1.25 and 1.13, which implies that Ni_2_P/rGO/NF has more defects than NiO/rGO/NF.

**Figure 4:**
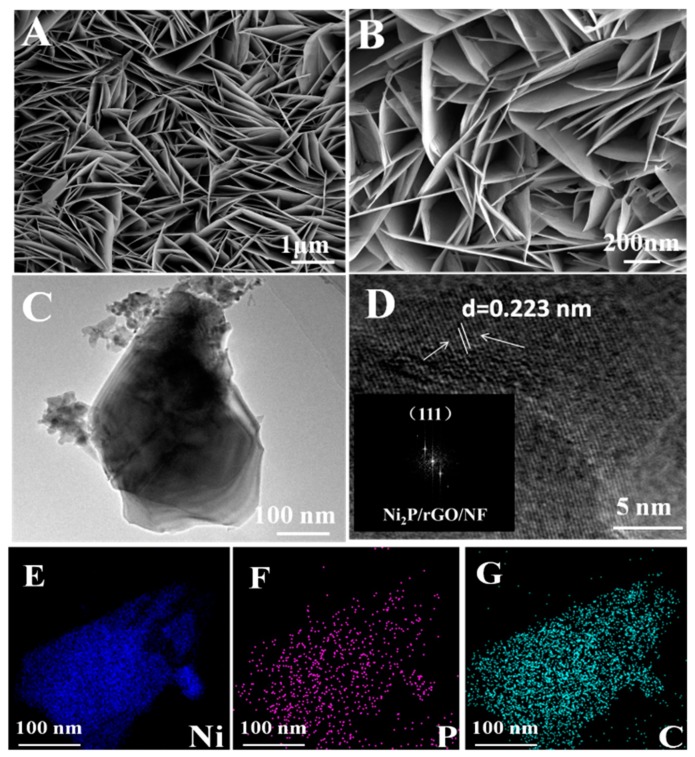
(**A**,**B**) Scanning Electron Microscopy (SEM); (**C**) Transmission Electron Microscopy (TEM); (**D**) High-Resolution Transmission Electron Microscopy (HRTEM) (inset: fast fourier transform (FFT) of Ni_2_P/rGO/NF); and (**E**–**G**) Element mapping of Ni_2_P/rGO/NF.

SEM helped to observe the morphology exhibited by these as-prepared catalysts. Based on the SEM image about Ni_2_P/rGO/NF, Ni foam (Figure S2A) is overspreaded with Ni_2_P/rGO nanosheets array (Figure 4A). It is clearly observed that the Ni_2_P/rGO/NF presents a shaggy and uniform sheet distribution (Figure 4B) and NiO/rGO/NF exhibits a sheet distribution (Figure S2B). The TEM about Ni_2_P/rGO/NF is displayed in Figure 4C, with sheet structure being detected clearly. Based on Figure 4D, crystal stripes can be found in the High-Resolution Transmission Electron Microscopy (HRTEM) of Ni_2_P/rGO/NF. Besides, inter lattice distance of 0.223 nm corresponds to Ni_2_P/rGO/NF (111) crystal plane and the fast fourier transform (FFT) of Ni_2_P/rGO/NF with distinct diffraction points and indicates a highly crystalline structure [35]. This phenomenon supports the XRD result. The elemental mappings display the uniform distribution of Ni (Figure 4E), P (Figure 4F) and C (Figure 4G) elements across the layer structure, which conforms that nickel phosphide nanosheets and rGO are successfully fabricated on the Ni foam.

### 3.2. Electrochemical Characterization

**Figure 5:**
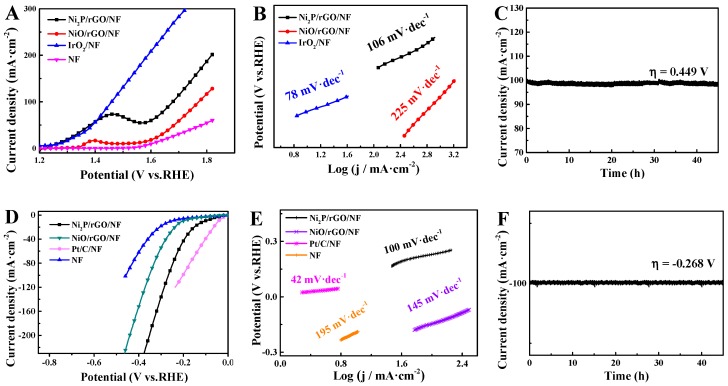
(**A**) LSV of Ni_2_P/rGO/NF, NiO/rGO/NF, IrO_2_/NF and NF and (**B**) Tafel slope of Ni_2_P/rGO/NF, NiO/rGO/NF, IrO_2_/NF and NF. (**C**) Multi-voltage process of Ni_2_P/rGO/NF. (**D**) LSV of Ni_2_P/rGO/NF, NiO/rGO/NF, IrO_2_/NF and NF and (**E**) Tafel slope of Ni_2_P/rGO/NF, NiO/rGO/NF, IrO_2_/NF and NF. (**F**) Chronoamperometry testing (i-t) about the Ni_2_P/rGO/NF.

Linear sweep voltammetry (LSV) of Ni_2_P/rGO/NF (loading: 0.35 mg/cm^2^) has been examined in various electrolytes towards OER (Figure 5A). At 100 mA/cm^2^, the overpotentials of Ni_2_P/rGO/NF, NiO/rGO/NF, as well as IrO_2_/NF are 449 mV, 542 mV and 230 mV respectively. Obviously, Ni_2_P/rGO/NF presents a lower overpotential compared with NiO/rGO/NF. It means that Ni_2_P/rGO/NF possesses a better OER activity. The electrode dynamics exhibited by the OER can be measured by virtue of the Tafel slope from LSVs [36]. The Tafel slopes of Ni_2_P/rGO/NF, NiO/rGO/NF and IrO_2_/NF are 106, 225, as well as 78 mV/dec, respectively (Figure 5B). Obviously, Ni_2_P/rGO/NF exhibits a lower Tafel slope than the monomer, meaning that its kinetics is fast and the OER catalytic activity is excellent [37]. Also, stability can greatly affect the OER. Based on Figure 5C, chronoamperometry testing (i-t) was conducted on Ni_2_P/rGO/NF at 0.449 V, finding that it can remain stable for no less than 44 hours. Following 3000 cycles, 92.2% of the initial value of current density can be kept (Figure S3) and XRD and SEM long-term stability is almost consistent with the previous test results (Figure S4). Hence, the stability is excellent [30].

We tested LSVs of Ni_2_P/rGO/NF in various electrolytes for analyzing its catalytic activity for HER. LSVs about the above prepared catalysts are displayed in Figure 5D. At 10 mA·cm^−2^, Ni_2_P/rGO/NF, NiO/rGO/NF, Pt/C/NF as well as NF exhibited an overpotential of 115, 151, 42 and 250 mV, respectively. The overpotential possessed by Ni_2_P/rGO/NF appears higher compared with Pt/C/NF but remains lower compared with NiO/rGO/NF and NF. The Tafel slopes of Ni_2_P/rGO/NF, NiO/rGO/NF, Pt/C/NF and NF reached 100 mV/dec, 145 mV/dec, 42 mV/dec and 195 mV/dec.

From Figure 5E, clearly, Ni_2_P/rGO/NF has a Tafel slope the nearest to Pt/C/NF, demonstrating its efficiency for the HER. Furthermore, HER was achieved in alkaline solution following the Tafel–Volmer–Heyrosky mechanism (Equations (1)–(3)) [38]. The crucial role of the formation of transition (M-H*) is the rate determining step (Tafel–Volmer step, Equation (3)) [39]. Ni_2_P/rGO/NF has excellent HER performance, which can be attributed to the advantageous Tafel-Volmer step. This promotes the release of low overpotential hydrogen.

M + H_2_O + e− → M − H* + OH^−^ (aq) (Volmer) (1)

M-H* + H_2_O + e− → M + H_2_ + OH^−^ (aq) (Heyrovsky) (2)

2M − H* → H_2_ + 2M (Tafel) (3)

Chronoamperometry testing (i–t) was implemented on Ni_2_P/rGO/NF at -0.268 V, showing that it is able to remain stable for no less than 44 h (Figure 5F). 

**Figure 6:**
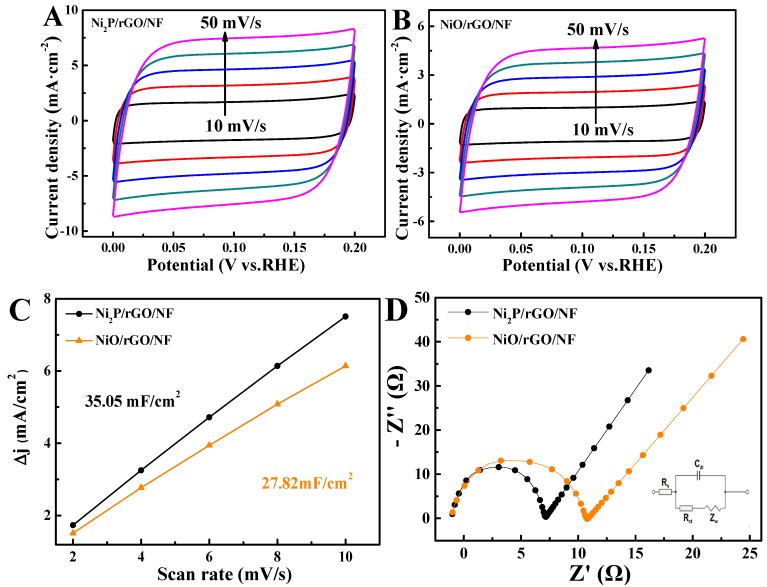
CV of (**A**) Ni_2_P/rGO/NF and (**B**) NiO/rGO/NF. (**C**) A plot of Δj and (**D**) EIS of Ni_2_P/rGO/NF and NiO/rGO/NF.

The slope of the electric double layer capacitance (Cdl) as a function of scan rate and Δj (j_a_-j_c)_, which reflects the changing trend of electrochemically active area (ECSA) by CV curves. The curves from the inside to the outside indicate that CV tests are performed at the scan rates 10, 20, 30, 40, and 50 mV/s, respectively. (Figure 6A,B). The measured potential range is 0 to 0.2 V without obvious redox regime. Cdl of 35.05 and 27.82 mF/cm^2^ is obtained for Ni_2_P/rGO/NF and NiO/rGO/NF (Figure 6C). Obviously, Cdl of Ni_2_P/rGO/NF is larger than that of NiO/rGO/NF indicates ECSA is larger, which reflects a large roughness and excellent HER activity [40,41]. Electrochemical impedance spectrum (EIS) were employed to further evaluate the electron transfer kinetics of all composites. Figure 6D shows Nyquist plots of Ni_2_P/rGO/NF and NiO/rGO/NF. The high-frequency semicircle represents charge transfer resistance (Rct). Obviously, the impedance of Ni_2_P/rGO/NF is lower to that of NiO/rGO/NF. Therefore, Ni_2_P/rGO/NF has excellent electron transfer rate [42].

**Figure 7:**
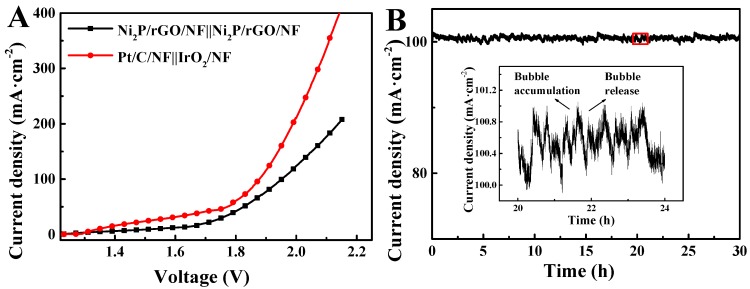
(**A**) LSVs of water electrolysis. (**B**) i-t testing of Ni_2_P/rGO/NF||Ni_2_P/rGO/NF.

A two-electrode electrolyzer, that is, Ni_2_P/rGO/NF||Ni_2_P/rGO/NF, was performed taking Ni_2_P/rGO/NF as the anode and the cathode for exploring the electrochemical performance exhibited by the Ni_2_P/rGO/NF electrode in the entire water splitting. And IrO_2_/NF as cathode and Pt/C/NF as anode (Pt/C/NF||IrO_2_/NF) are used to compare. Based on Figure 7A, Pt/C/NF||IrO_2_/NF requires voltages of 1.502 V at 10 mA/cm^2^. While Ni_2_P/rGO/NF||Ni_2_P/rGO/NF needs the value of 1.676 V. As indicated, Ni_2_P/rGO/NF||Ni_2_P/rGO/NF electrolyzer has a close performance to that of Pt/C/NF||IrO_2_/NF. Also, the study compared the electochemical performance exhibited by these materials in literature, finding that the catalytic activity possessed by Ni_2_P/rGO was more excellent compared with other materials (Tables S1 and S2). In addition, chronoamperometry (i-t) measurement was carried out under 1.919 V applied voltage on the water splitting, demonstrating the ability of Ni_2_P/rGO/NF||Ni_2_P/rGO/NF to preserve a higher current density within more than 30 h of operation (Figure 7B). Therefore, it has a strong stability. The inset of Figure 7C shows the fluctuation of current density around the 22th hours, which may be caused by the accumulation and release of the remaining bubbles on the electrode surface [43].

## 4. Conclusions

In summary, Ni_2_P/rGO/NF acts as a metal catalyst with a high efficiency and strong stability for OER. The overpotential of Ni_2_P/rGO/NF reaches 1.679 V at 100 mA/cm^2^. Besides, Ni_2_P/rGO/NF presents a high level of activity to HER and only 110 mV is needed for achieving 10 mA/cm^2^. On that account, Ni_2_P/rGO/NF serves as a bifunctional electrocatalytic material for the OER and the HER. The Ni_2_P/rGO/NF||Ni_2_P/rGO/NF has the function of driving 10 mA/cm^2^ at 1.676 V and can maintain electrolysis for at least 30 hours. The study helps to understand the hydrogen production via the transition metal nitride nanoarrays from a new perspective.

## Supplementary Materials

The following are available online at https://www.mdpi.com/1996-1944/13/3/744/s1, Figure S1: X-ray Diffraction (XRD) pattern of NiO/rGO/NF. Figure S2. (A) Scanning Electron Microscopy (SEM) image of bare Ni foam. (B) SEM image of NiO/rGO/NF. Figure S3. LSV curves of Ni2P/rGO/NF for 3000 cycles before and after operation, respectively Figure S4. (A) XRD image of Ni2P/rGO/NF and (B) SEM image of Ni2P/rGO/NF after long-term stability. Table S1. Comparison of the HER activity for several recently reported catalysts. Table S2. Comparison of the oxygen evolution reaction (OER) activity for several recently reported catalysts.

## References

[1] Khajehsaeidi Z., Sangpour P., Ghaffarinejad A. (2019). A novel co-electrodeposited Co/MoSe_2_/reduced graphene oxide nanocomposite as electrocatalyst for hydrogen evolution. Int. J. Hydrog. Energy.

[2] Anantharaj S., Ede S.R., Karthick K., Sankar S.S., Sangeetha K., Karthik P.E., Kundu S. (2018). Precision and correctness in the evaluation of electrocatalytic water splitting: Revisiting activity parameters with a critical assessment. Energy Environ. Sci..

[3] You H., Wu Z., Jia Y., Xu X., Xia Y., Han Z., Wang Y. (2017). Highefficiency and mechano-/photo-bi-catalysis of piezoelectricZnO@photoelectric-TiO_2_ core-shell nanofibers for dye decomposition. Chemosphere.

[4] Zhou H.Q., Yu F., Zhu Q., Sun J.Y., Qin F., Yu L., Bao J.M., Yu Y., Chen S., Ren Z.F. (2018). Water splitting by electrolysis at high current densities under 1.6volts. Energy Environ. Sci..

[5] You B., Sun Y. (2018). Innovative strategies for electrocatalytic water splitting. Acc. Chem. Res..

[6] Li Y.C., He B., Liu X.Q., Hu X.Q., Huang J., Ye S.Q., Zhu S., Yang W., Zhen L. (2019). Graphene confined MoS_2_ particles for accelerated electrocatalytic hydrogen evolution. Int. J. Hydrog. Energy.

[7] Zhu M., Sun Z., Fujitsuka M., Majima T. (2018). Z-Scheme Photocatalytic Water Splitting on a 2D Heterostructure of Black Phosphorus/Bismuth Vanadate Using Visible Light. Angew. Chem. Int. Edit..

[8] Nam S., Mai C.T.K., Oh I. (2018). Ultrastable Photoelectrodes for Solar Water Splitting Based on Organic Metal Halide Perovskite Fabricated by Lift-Off Process. ACS Appl. Mater. Inter..

[9] Zhang W., Yan D., Tong X., Liu M. (2018). Ultrathin Lutetium Oxide Film as an Epitaxial Hole-Blocking Layer for Crystalline Bismuth Vanadate Water Splitting Photoanodes. Adv. Funct. Mater..

[10] Tan C., Cao X., Wu X.J., He Q., Yang J., Zhang X., Chen J., Zhao W., Han S., Nam G.H. (2017). Recent Advances in Ultrathin Two-Dimensional Nanomaterials. Chem. Rev..

[11] Wang J., Cui W., Liu Q., Xing Z., Asiri A.M., Sun X. (2016). Recent Progress in Cobalt-based Heterogeneous Catalysts for Electrochemical Water Splitting. Adv. Mater..

[12] Voiry D., Yang J., Chhowalla M. (2016). Recent Strategies for Improving the Catalytic Activity of 2D TMD Nanosheets toward the Hydrogen Evolution Reaction. Adv. Mater..

[13] Liang Y., Liu Q., Asiri A.M., Sun X., Luo Y. (2014). Self-supported FeP Nanorod Arrays: A Cost-effective 3D Hydrogen Evolution Cathode with High Catalytic Activity. ACS Catal..

[14] Yang J., Wang K., Zhu J., Zhang C., Liu T. (2016). Self-templated Growth of Vertically Aligned 2H-1T MoS_2_ for Efficient Electrocatalytic Hydrogen Evolution. ACS Appl. Mater. Inter..

[15] Nitin K.C., Haneul J., Byeongyoon K., Lee K. (2017). Nanostructured Materials on 3D Nickel Foam as Electrocatalysts for Water Splitting. Nanoscale.

[16] Song L., Kang X., Zhang S. (2018). CNT/g-C_3_N_4_ photocatalysts with enhanced hydrogen evolution ability for water splitting based on a noncovalent interaction. Int. J. Energy Res..

[17] Hu Y.P., Li F., Long Y., Yang H.D., Gao L.L., Long X.F., Hu H.G., Xu N., Jin J., Ma J.T. (2018). Ultrafine CoPS nanoparticles encapsulated in N, P, and S tri-doped porous carbon as an efficient bifunctional water splitting electrocatalyst in both acid and alkaline solutions. J. Mater. Chem. A.

[18] Weng B.C., Grice C.R., Meng W.W., Guan L., Xu F.H., Yu Y., Wang C.L., Zhao D.W., Yan Y.F. (2018). Metal–Organic Framework-Derived CoWP@C Composite Nanowire Electrocatalyst for Efficient Water Splitting. ACS Energy Lett..

[19] Kong D.S., Cha J.J., Wang H.T., Lee H.R., Cui Y. (2013). First-Row Transition Metal Dichalcogenide Catalysts for Hydrogen Evolution Reaction. Energy Environ. Sci..

[20] Wang B., Wang Z., Wang X., Zheng B., Zhang W., Chen Y. (2018). Scalable Synthesis of Porous Hollow CoSe_2_–MoSe_2_/Carbon Microspheres for Highly Efficient Hydrogen Evolution Reaction in Acidic and Alkaline Media. J. Mater. Chem. A.

[21] Zheng B.J., Chen Y.F., Qi F., Wang X.Q., Zhang W.L., Li Y.R., Li X.S. (2017). 3D-Hierarchical MoSe_2_ Nanoarchitecture as a Highly Efficient Electrocatalyst for Hydrogen Evolution. 2D Mater..

[22] Popczun E.J., McKone J.R., Read C.G., Biacchi A.J., Wiltrout A.M., Lewis N.S., Schaak R.E. (2013). Nanostructured Nickel Phosphide as an Electrocatalyst for the Hydrogen Evolution Reaction. J. Am. Chem. Soc..

[23] Kannan R., Kim A.R., Nahm K.S., Lee H.K., Jin Yoo D. (2014). Synchronized synthesis of Pd@C-RGO carbocatalyst for improved anode and cathode performance for direct ethylene glycol fuel cell. Chem. Commun..

[24] Wang X.Q., Zheng B.J., Yu B., Wang B., Hou W.Q., Zhang W.L., Chen Y.F. (2018). In-Situ Synthesis of Hierarchical MoSe_2_-CoSe_2_ Nanotubes as Efficient Electrocatalyst for Hydrogen Evolution Reaction in both Acidic and Alkaline Medium. J. Mater. Chem. A.

[25] Yu B., Qi F., Chen Y.F., Wang X.Q., Zheng B.J., Zhang W.L., Li Y.R., Zhang L.C. (2017). Nanocrystalline Co_0.85_Se Anchored on Graphene Nanosheets as a Highly Efficient and Stable Electrocatalyst for Hydrogen Evolution Reaction. ACS Appl. Mater. Inter..

[26] Yang Y.Q., Zhang K., Lin H.L., Li X., Chan H.C., Yang L.C., Gao Q.S. (2017). MoS_2_–Ni_3_S_2_ Heteronanorods as Efficient and Stable Bifunctional Electrocatalysts for Overall Water Splitting. ACS Catal..

[27] Yan Y., Xia B.Y., Ge X.M., Liu Z.L., Wang J.Y., Wang X. (2013). Ultrathin MoS_2_ Nanoplates with Rich Active Sites as Highly Efficient Catalyst for Hydrogen Evolution. ACS Appl. Mater. Inter..

[28] Ravikumar C.H., Nair G.V., Muralikrishna S., Nagaraju D.H., Balakrishna R.G. (2018). Nanoflower like Structures of MoSe_2_ and MoS_2_ as Efficient Catalysts for Hydrogen Evolution. Mater. Lett..

[29] Tang C., Zhang R., Lu W.B., Wang Z., Liu D.N., Hao S., Du G., Asiri A.M., Sun X.P. (2016). Energy-Saving Electrolytic Hydrogen Generation: Ni_2_P Nanoarray as a High-Performance Non-Noble-Metal Electrocatalyst. Angew. Chem. Int. Edit..

[30] Hu S.N., Feng C.Q., Wang S.Q., Liu J.W., Wu H.M., Zhang L., Zhang J.J. (2019). Ni_3_N/NF as Bifunctional Catalysts for Both Hydrogen Generation and Urea Decomposition. ACS Appl. Mater. Inter..

[31] Yang L.Q., Liu Y.L., Wang L., Zhao Z.J., Xing C.J., Shi S.H., Yuan M.L., Ge Z.M., Cai Z.Y. (2019). Co_5.47_N/rGO@NF as a High-Performance Bifunctional Catalyst for Urea-Assisted Hydrogen Evolution. Catal. Lett..

[32] Yu B., Wang X., Qi F., Zheng B., He J., Lin J., Zhang W., Li Y., Chen Y. (2017). Self-Assembled Coral-like Hierarchical Architecture Constructed by NiSe_2_ Nanocrystals with Comparable Hydrogen-Evolution Performance of Precious Platinum Catalyst. ACS Appl. Mater. Interf..

[33] Wu M.Y., Da P.F., Zhang T., Mao J., Liu H., Ling T. (2018). Designing Hybrid NiP_2_/NiO Nanorod Arrays for Efficient Alkaline Hydrogen Evolution. ACS Appl. Mater. Interf..

[34] Min S.D., Zhao C.G., Chen G.R., Qian X.Z. (2014). One-Pot Hydrothermal Synthesis of Reduced Graphene Oxide/Ni(OH)_2_ Films on Nickel Foam for High Performance Supercapacitors. Electrochim. Acta.

[35] Song R., Luo B., Geng J., Song D., Jing D. (2018). Photothermocatalytic Hydrogen Evolution over Ni_2_P/TiO_2_ for Full-Spectrum Solar Energy Conversion. Ind. Eng. Chem. Res..

[36] Kuai C.G., Zhang Y., Wu D.Y., Sokaras D., Mu L.Q., Spence S., Nordlund D., Lin F., Du X.W. (2019). Fully Oxidized Ni-Fe Layered Double Hydroxide with 100% Exposed Active Sites for Catalyzing Oxygen Evolution Reaction. ACS Catal..

[37] Zhu Y.H., Xu Z.W., Yan K., Zhao H.B., Zhang J.D. (2017). One-Step Synthesis of CuO–Cu_2_O Heterojunction by Flame Spray Pyrolysis for Cathodic Photoelectrochemical Sensing of l-Cysteine. ACS Appl. Mater. Inter..

[38] Qiao L.Q., Zhou M., Li Y.H., Zhang A.J., Deng J., Liao M.J., Xiao P., Zhang Y.H., Zhang S.T. (2014). Enhancing Electrochemical Hydrogen Generation by Platinum-Modification of p-Type Silicon Wires Array under Visible Light. J. Electrochem. Soc..

[39] Sivanantham A., Ganesan P., Shanmugam S. (2016). Bifunctional Electrocatalysts: Hierarchical NiCo_2_S_4_ Nanowire Arrays Supported on Ni Foam: An Efficient and Durable Bifunctional Electrocatalyst for Oxygen and Hydrogen Evolution Reactions. Adv. Funct. Mater..

[40] Wang J.M., Ma X., Qu F.L., Asiri A.M., Sun X.P. (2017). Fe-doped Ni_2_P nanosheet array for high-efficiency electrochemical water oxidation. Inorg. Chem..

[41] Jiang J., Gao M.R., Sheng W.C., Yan Y.S. (2016). Hollow chevrel-phase NiMo3S4 for hydrogen evolution in alkaline electrolytes. Angew. Chem. Int. Ed..

[42] Jia D.D., Gao H.Y., Xing L.W., Chen X., Dong W.J., Huang X.B., Wang G. (2019). 3D Self-Supported Porous NiO@NiMoO_4_ Core–Shell Nanosheets for Highly Efficient Oxygen Evolution Reaction. Inorg. Chem..

[43] Zhang Y., Wang Y.H., Jia S.P., Xu H.Q., Zang J.B., Liu J., Xu X.P. (2016). A hybrid of NiMo-Mo_2_C/C as non-noble metal electrocatalyst for hydrogen evolution reaction in an acidic solution. Electrochim. Acta.

