# Supplementary material for: Ni2P/rGO/NF Nanosheets As a Bifunctional High-Performance Electrocatalyst for Water Splitting
Source: Materials (Basel). 2020 Feb 6;13(3):744. doi: 10.3390/ma13030744 (PMC7041371; doi:10.3390/ma13030744)
Supplement: Supplementary file 1 [file materials-13-00744-s001.pdf]

Supplementary Materials: Ni_2P/rGO/NF Nanosheets as a Bifunctional High-Performance Electrocatalyst for Water Splitting

Jinyu Huang, Feifei Li, Baozhong Liu and Peng Zhang *

1. XRD pattern of NiO/rGO/NF

Figure S1 shows the X-ray Diffraction (XRD) pattern of NiO/rGO/NF. Obviously, the diffraction peaks at 37.18°, 43.22°, and 62.82° are indexed to the (111), (200), and (220) of NiO (JCPDS No. 42-1300). The peaks at 44.51° and 51.82° belonged to (111) and (200) of Ni (JCPDS No. 04-0850), which are from NF. While the peak at 20.04° is derived from reduced graphene oxide.

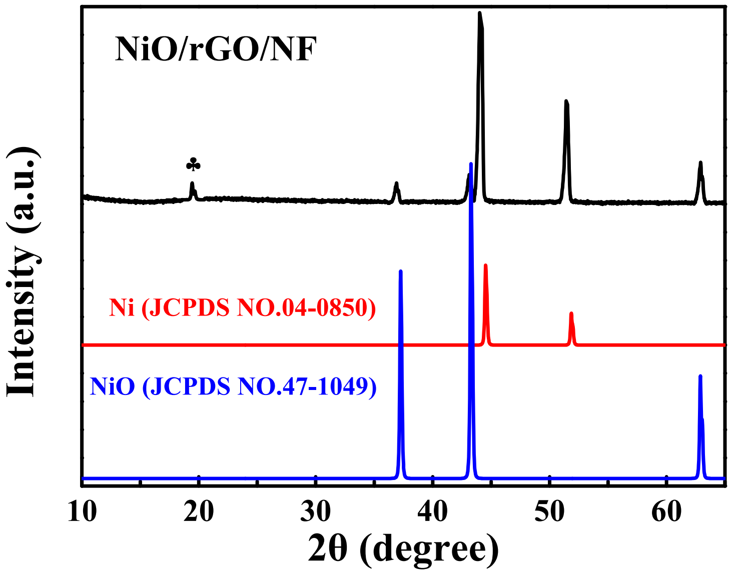

Figure S1. X-ray Diffraction (XRD) pattern of NiO/rGO/NF.

2. SEM Images of Bare Ni Foam and NiO/rGO/NF

Figure S2 displays the Scanning Electron Microscopy (SEM) image of bare NF, indicating the surface is smooth. The SEM images of NiO/rGO/NF (Figure S2B) reveals the gracile nanosheets arrays grow uniformly on NF.

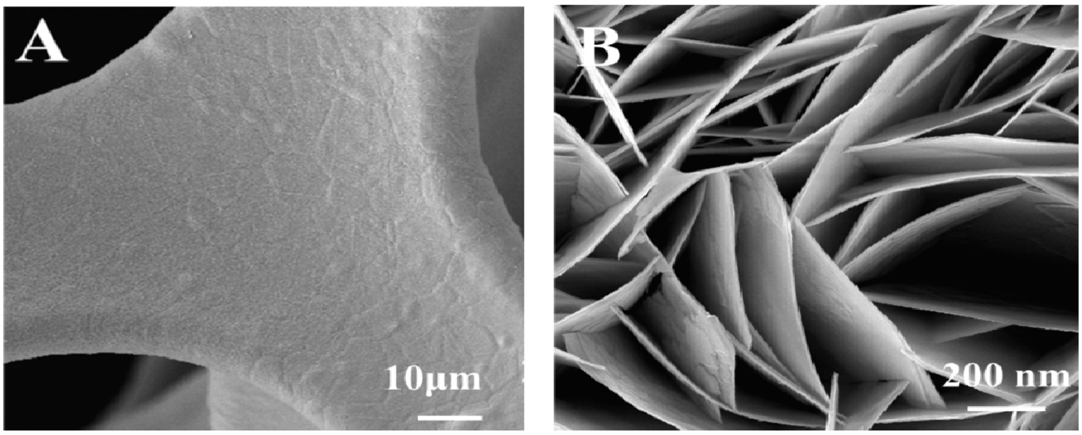

Figure S2. (**A**) Scanning Electron Microscopy (SEM) image of bare Ni foam. (**B**) SEM image of NiO/rGO/NF.

3. LSV Curves of Ni_2P/rGO/NF.

The current density could retain 92.2% of the initial value after 3000 cycles (Figure S3). This results indicate the excellent stability of Ni_2P/rGO/NF catalyst.

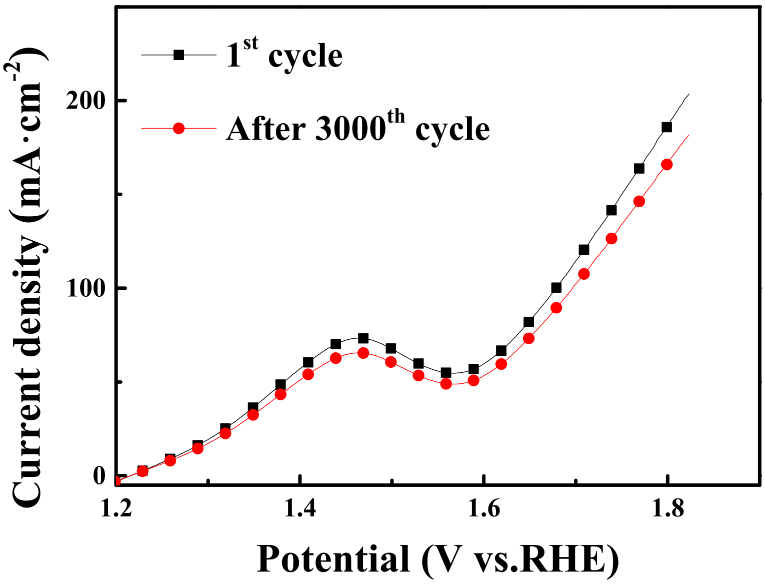

Figure S3. LSV curves of Ni_2P/rGO/NF for 3000 cycles before and after operation, respectively

4. XRD and SEM Images of Ni_2P/rGO/NF after Long-Term Stability.

The long-term stability means that Ni_2P/rGO/NF is continuously scanned 3000 cyclic voltammetry (CV) cycles in HER. Figure S4A displays the XRD image of Ni_2P/rGO/NF after long-term stability, indicating the slight change in structure. The SEM images of Ni_2P/rGO/NF after long-term stability (Figure S4B) reveals the gracile nanosheets arrays grow uniformly on NF and the structure of sheet is the same as before 3000 CV cycles (Figure 4B). This implies that Ni_2P/rGO/NF has a superior stability.

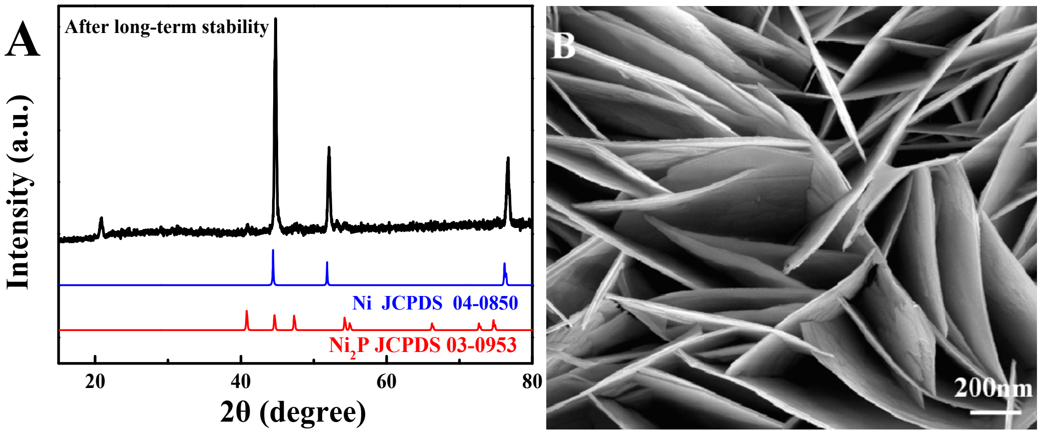

Figure S4. (**A**) XRD image of Ni_2P/rGO/NF and (**B**) SEM image of Ni_2P/rGO/NF after long-term stability.

5. Comparison of Catalytic Performance of some HER Catalysts in Recent Years

The catalytic performance of catalysts for hydrogen evolution reaction (HER) in the literature is compared with our catalyst, as shown in Table S1. Obviously, the electrode potential of Ni_2P/rGO/NF is lower than those of other HER catalysts.

Table S1. Comparison of the HER activity for several recently reported catalysts.

Catalyst	Current Density (j, mA cm^{-2})Geometrical	η at the Corresponding j (mV)	Reference
Ni_2P/rGO/NF	10	115	This work
U-CNT-900	10	255	1
Co-NCNT/CC[a]	10	180	2
Ni_2P nanoparticles	10	230	3
WP_2 submicroparticles	10	153	4
CoP/CC[a]	10	209	5

6. Comparison of Catalytic Performance of some OER Catalysts in Recent Years

Comparing the catalytic performance of catalysts for oxygen evolution reaction (OER) in recent years. Obviously, we can discover that the potential of the materials we prepared are lower than that of most OER catalysts in 1M KOH. This implies Ni_2P/rGO/NF has superior catalytic activity and greater commercial possibilities.

Table S2. Comparison of the oxygen evolution reaction (OER) activity for several recently reported catalysts.

Catalyst	Current Density (j, mA cm^{-2}) Geometrical	η at the Corresponding j (mV)	Reference
Ni_2P/rGO/NF	50	330	This work
Ti-TiO_2-IrO_2-RuO_2	50	542	6
N doped etched Stainless steel (NESS)	50	350	7
CoO/NF	50	422	8
NiO@$NiMoO_4$ submicroparticles	50	335	9
Ni-doped CoS_2-2/CF	50	358	10

References

1. Gao, S.; Li, G.-D.; Liu, Y.; Chen, H.; Feng, L.-L.; Wang, Y.; Yang, M.; Wang, D.; Wang, S.; Zou, X. Electrocatalytic H2production from seawater over Co, N-codoped nanocarbons. *Nanoscale* **2015**, *7*, 2306–2316.
2. Xing, Z.; Liu, Q.; Xing, W.; Asiri, A.M.; Sun, X. Interconnected Co-Entrapped, N-Doped Carbon Nanotube Film as Active Hydrogen Evolution Cathode over the Whole pH Range. *ChemSusChem* **2015**, *8*, 1850–1855.
3. Feng, L.; Vrubel, H.; Bensimon, M.; Hu, X. Easily-prepared dinickel phosphide (Ni2P) nanoparticles as an efficient and robust electrocatalyst for hydrogen evolution. *Phys. Chem. Chem. Phys.* **2014**, *16*, 5917.
4. Xing, Z.C.; Liu, Q.; Asiri, A.M.; Sun, X.P. High-Efficiency electrochemical hydrogen evolution catalyzed by tungsten phosphide submicroparticles. *ACS Catal.* **2015**, *5*, 145–149.
5. Tian, J.; Liu, Q.; Asiri, A.M.; Sun, X. Self-Supported Nanoporous Cobalt Phosphide Nanowire Arrays: An Efficient 3D Hydrogen-Evolving Cathode over the Wide Range of pH 0–14. *J. Am. Chem. Soc.* **2014**, *136*, 7587–7590.
6. Maruthapandian, V.; Muthurasu, A.; Dekshinamoorthi, A.; Aswathy, R.; Vijayaraghavan, S.; Muralidharan, S.; Saraswathy, V.; Viruthasalam, M.; Amuthan, D.; Saranyan, V. Electrochemical Cathodic

Treatment of Mild Steel as a Host for Ni(OH) 2 Catalyst for Oxygen Evolution Reaction in Alkaline Media. *ChemElectroChem* **2019**, *6*, 4391–4401.

7. Park, S.; Khan, Z.; Shin, T.J.; Kim, Y.; Ko, H. Rechargeable Na/Ni batteries based on the Ni(OH)2/NiOOH redox couple with high energy density and good cycling performance. *J. Mater. Chem. A* **2019**, *7*, 1564–1573.
8. Zhu, S.; Lei, J.; Qin, Y.; Zhang, L.; Lu, L. Spinel oxide CoFe2O4 grown on Ni foam as an efficient electrocatalyst for oxygen evolution reaction. *RSC Adv.* **2019**, *9*, 13269–13274.
9. Du, J.; Zou, Z.; Liu, C.; Xu, C. Hierarchical Fe-doped Ni3Se4 ultrathin nanosheets as an efficient electrocatalyst for oxygen evolution reaction. *Nanoscale* **2018**, *10*, 5163–5170.
10. Xie, Z.; Tang, H.; Wang, Y. MOF-Derived Ni-Doped CoS 2 Grown on Carbon Fiber Paper for Efficient Oxygen Evolution Reaction. *ChemElectroChem* **2019**, *6*, 1206–1212.

© **2020** by the authors. Licensee MDPI, Basel, Switzerland. This article is an open access article distributed under the terms and conditions of the Creative Commons Attribution (CC BY) license (http://creativecommons.org/licenses/by/4.0/).
